# Association between carotid intima–media thickness and acute kidney injury following isolated coronary artery bypass surgery

**DOI:** 10.5830/CVJA-2022-035

**Published:** 2023-08-01

**Authors:** Çağrı Düzyol, Hüseyin Şaşkin

**Affiliations:** Cardiovascular Surgery Clinic, Derince Training and Research Hospital, Health Sciences University, Kocaeli, Turkey

**Keywords:** coronary artery bypass grafting, acute kidney injury, carotid intima–media thickness

## Abstract

**Objective:**

The association between pre-operative carotid intima–media thickness (CIMT) and early postoperative acute kidney injury (AKI) following isolated coronary artery bypass grafting (CABG) was investigated.

**Methods:**

Data were sought retrospectively of 237 patients (166 male, 71 female; mean age 61.4 ± 8.1 years; range: 32–74), operated on for isolated CABG with cardiopulmonary bypass (CPB) in a single centre between June 2014 and December 2020, with a serum creatinine level < 1.5 mg/dl and normal carotid arteries on Doppler ultrasonography. AKI diagnosis was made according to the Kidney Disease Improving Global Outcomes 2012 Acute Kidney Injury Guideline. Patients were grouped as group 1 with AKI in the early postoperative period (n = 63) and group 2 without AKI (n = 174). Univariate analyses were done to determine significant clinical factors, and subsequent multiple logistic regression analysis was done to determine independent predictors of AKI.

**Results:**

AKI occurred in 63 (26.6%) patients. Pre-operative CIMT was significantly higher in the AKI group (p = 0.0001). Multivariate logistic regression analysis revealed that elevated pre-operative CIMT (p = 0.005), C-reactive protein (p = 0.001), erythrocyte sedimentation rate (p = 0.005), neutrophil– lymphocyte ratio (p = 0.0001) and platelet–lymphocyte ratio (p = 0.0001) increased on the postoperative seventh day. C-reactive protein (p = 0.04), postoperative first day platelet– lymphocyte ratio (p = 0.0001), postoperative seventh day erythrocyte sedimentation rate (p = 0.02) and intubation time (p = 0.02) were independent predictors of early postoperative AKI following isolated CABG.

**Conclusion:**

Pre-operative CIMT was found to be an independent predictor of AKI in the early postoperative period of isolated CABG.

Cardiovascular disease is the main cause of mortality and morbidity in Western and developing countries.^1^ Endothelial dysfunction is the key to arteriosclerosis with disruption of endothelial homeostasis predisposing to vasoconstriction, inflammation, leukocyte adhesion, thrombosis and vascular smooth muscle cell proliferation.^2^

The atherosclerotic process results in increased carotid intima–media thickness (CIMT) as early structural change.^3^ CIMT has been proposed as a quantitative index of subclinical atherosclerotic disease progression and surrogate measures for cardiovascular disease.^4^ Elevated CIMT was reported as an atherosclerosis marker related to myocardial infarction, stroke and peripheral arterial disease.^3^ Moreover, CIMT is a parameter used to estimate other conventional vascular risk factors and events.^5^ CIMT increases in high-risk populations such as the elderly with hypertension, diabetes mellitus and chronic kidney disease.^6^

As an important pathology, acute kidney injury (AKI) often follows cardiac surgery, resulting in extended intensive care unit (ICU) and hospital stays, and increased expenses and mortality rates.^7^ The reported incidence of AKI following cardiac surgery is 5–30%.^8^ Multiple factors contribute to postoperative AKI, including advanced age, female gender, delay between cardiac catheterisation and surgery, aortic cross clamping (ACC) and cardiopulmonary bypass (CPB) duration, intra-operative mean arterial pressure differences and postoperative blood transfusion.^9^ Impaired endothelial function plays a central role in the pathogenesis of AKI.^10^

Renal dysfunction is known as one of the cardiovascular risk factors or markers that may improve risk prediction and identification in high-risk patients.^4^ Various biomarkers have recently been used to diagnose AKI as one of the important causes of morbidity and mortality in the early postoperative period of coronary artery bypass grafting (CABG). To date, the most studied and promising biomarkers have been neutrophil gelatinase-associated lipocalin (NGAL), interleukin-18 (IL-18), kidney injury molecule-1 and cystatin C.^11^ Liangos et al. reported that peri-operative plasma IL-8 levels predicted the occurrence of postoperative AKI among adults operated on for CABG with CPB.^12^ Fanning et al. reported urinary NGAL, measured 24 hours after the initiation of CPB, to be a better predictor of postoperative AKI.^13^

Recently, neutrophil–lymphocyte ratio (NLR) and platelet– lymphocyte ratio (PLR) have been investigated as prognostic factors for cardiovascular disease, and their mutual role together with endothelial inflammation appeared as optimal predictors for major cardiovascular events and prognostic factors of postprocedural results.^14^ Parlar et al. reported that postoperative NLR in the first four days after CABG was a useful biomarker to predict AKI during in-hospital follow up.^15^ Elevated NLR and PLR in the pre- and postoperative periods of CABG were reported as independent biomarkers directly associated with early postoperative AKI.^16^

Cross-sectional studies on the general population showed an inversely proportional relationship between renal function and CIMT.^6^ Zhang et al. reported significantly higher levels of CIMT in patients with early-stage chronic kidney disease.^17^ There have been very few studies in the recent literature that examined the relationship between early postoperative AKI following isolated CABG and CIMT as an inflammatory marker. The case–control study by Onk et al. reported that common carotid artery IMT (CCA-IMT) measurements prior to CABG were significantly higher in patients with postoperative AKI.^18^

This study aimed to analyse markers of inflammation together with CIMT, search for associated risk factors of early postoperative AKI, and supply a base for clinical prevention and reduction of AKI following CABG.

## Methods

Retrospective data were analysed of 289 patients, operated on for isolated CABG with CPB in the same centre by the same team between June 2014 and December 2020, whose carotid artery systems were normal on Doppler ultrasonography and CIMT was measured in the pre-operative period. Among these, we enrolled 237 patients who were diagnosed with critical coronary artery disease (≥ 75% narrowing) and operated on for CABG with CPB, and who had a pre-operative serum creatinine level < 1.5 mg/dl.

A diagnosis of AKI was made by comparing baseline and postoperative serum creatinine (sCr) levels to determine the predefined significant change based on the Kidney Disease Improving Global Outcomes (KDIGO) definition (increase in sCr level by ≥ 0.3 mg/dl within 48 hours of surgery or increase in sCr to ≥ 1.5 times baseline within three days of cardiac surgery).^19^ AKI diagnosis was based on the highest sCr concentration measured during the first three postoperative days compared with the baseline sCr concentration, defined as the last concentration before surgery. Urine output, affected by diuretics administered during anaesthesia and CPB, was not used to define AKI. Patients who developed AKI in the early postoperative period were classified as group 1 (n = 63) and those with normal postoperative renal function as group 2 (n = 174).

We excluded patients previously diagnosed with end-stage renal disease and on dialysis. We also excluded patients who had peripheral or carotid arterial disease, valvular heart disease, chronic obstructive pulmonary disease, malignancy, endocrinological disorders, advanced age (> 75 years), systemic inflammatory diseases, left ventricular ejection fraction (LVEF) ≤ 30%, decompensated congestive heart failure, congenital cardiac disease, severely overweight (body mass index > 30 kg/m^2^), renal impairment (sCr > 1.5 mg/dl), haematological proliferative diseases, low pre-operative haemoglobin (≤ 10 g/dl), on steroid or non-steroidal anti-inflammatory drugs, pre-operative immunosuppressive drug treatment within the last two weeks, the presence of signs of clinical infection [fever > 37.5°C, C-reactive protein (CRP) ≥ 5 mg/dl, erythrocyte sedimentation rate (ESR) > 20 mm/h or leukocyte count > 11 000 cells/μl] pre-operatively, patients with acute myocardial infarction and percutaneous coronary intervention in the last 30 pre-operative days, emergent operations, re-operations due to haemodynamic instability or bleeding, intra-aortic balloon pump requirement, operations on a beating heart or redo CABG.

Proximal aortic anastomosis sites were examined by palpation, since we did not perform routine pre-operative computed tomography. Patients ineligible for side clamping, whose proximal anastomoses were done in a single ACC period, were excluded. Patients with missing data such as sCr level or urine output and whose cardiac catheterisations were performed within the last 15 pre-operative days were also excluded.

Carotid artery ultrasonography and CIMT measurements were performed using the M Turbo ultrasonography device with a 5–12-MHz superficial probe. Carotid imaging was performed in the supine position with an approximate contralateral neck angle of 20°. Different measurements of CIMT were obtained from the common carotid arteries, bifurcations and the first 2 cm of the internal carotid arteries bilaterally, and the posterior walls were only evaluated. These measurements were performed with B-mode on the longitudinal axis from the level between the luminal and media/adventitia echogenities. The average of CIMT measured three times on both carotid arteries was calculated.^20^

The demographic and clinical data of the patients were obtained from the hospital software system. Age, gender, smoking status, diabetes, hypertension, hyperlipidaemia, LVEF, laboratory parameters, operation information, the number of grafts used, duration of CPB and ACC, use of blood products and length of ICU and hospital stays were recorded. PLR was calculated by dividing the number of platelets by the number of lymphocytes. NLR was calculated by dividing the number of neutrophils by the number of lymphocytes.

This study complied with the Declaration of Helsinki and was carried out following approval of the Ethics Committee for Clinical Trials of Kocaeli Derince Training and Research Hospital of Health Sciences University (Approval date no: 28.01.2021- 2021/19).

All patients were operated on with a median sternotomy under general anaesthesia. Standard CPB was established with aortic and venous cannulations, and systemic heparin (300 IU/ kg) with the maintenance of activated clotting time (ACT) > 450 seconds. Hyperkalaemic cold blood cardioplegia was applied for cardiac arrest. Surgery was performed under moderate hypothermia (28–30oC). CPB flow was maintained at 2.2–2.5 l/ min/m^2^, mean perfusion pressure between 50 and 80 mmHg, and haematocrit level between 20 and 25%. All distal anastomoses were done during the ACC period and the proximal anastomoses onto the ascending aorta on a beating heart. All patients were extubated following the onset of spontaneous breathing, normalised co-operation and appropriate haemodynamic and respiratory function.

## Statistical analysis

Statistical analysis was performed using the SPSS software version 22.0. Among the data measured, those showing normal distribution are expressed as mean ± standard deviation; those not showing a normal distribution are expressed as median (minimum–maximum). The data obtained by counting are given as percentages. Among the data measured, the difference between the groups was evaluated by the Student’s t-test in normal and homogenous distribution, and by the Mann–Whitney U-test in distribution that was not normal and homogenous. Among the data obtained by counting, the differences between the groups were evaluated by the parametric or non-parametric Pearson’s chi-squared test or Fisher’s exact test according to the distribution being parametric or not.

The effects of the risk factors suggested to be influential on the early-period AKI were studied with univariate logistic regression analysis. The multiple effects of the risk factors that are effective or suggested to be effective in predicting early postoperative AKI, as a result of univariate statistical analysis, were studied with retrospective selective multivariate logistic regression analysis. The odds ratio, 95% confidence interval and significance level for each of the risk factors were found. The sensitivity and specificity of CIMT in predicting early postoperative AKI via receiver operating characteristic (ROC) curves were computed and results were found statistically significant for p < 0.05.

## Results

The demographic characteristics and clinical data of the patients are summarised in Table 1. CIMTs were statistically significantly higher in group 1 patients (p = 0.0001). There were no statistically significant differences between the groups in terms of demographic and clinical properties.

**Table 1:** Comparison of patients’ characteristics between the two groups

*Patients' characteristics*	*Group I AKI (n = 63)*	*Group II non-AKI (n = 174)*	*p-value*
Age (years), median (min-max)	63 (42-74)	61.7 (32-74)	0.17**
Male, n (%)	47 (74.6)	119 (68.4)	0.36*
Female, n (%)	16 (25.4)	55 (31.6)	
BMI (kg/m²), median (min-max)	26.7 (20.5-33.7)	26.3 (18.3-37.1)	0.53**
Hypertension, n (%)	21 (33.3)	57 (32.8)	0.93*
Diabetes mellitus, n (%)	22 (34.9)	65 (37.4)	0.73*
Smoking, n (%)	28 (44.4)	58 (33.302)	0.12*
Hyperlipidaemia, n (%)	26 (41.3)	79 (45.4)	0.57*
Ejection fraction (%), median (min-max)	55.8 (35-70)	55.6 (35-72)	0.87**
Basal heart rate, median (min-max)	66.4 (55-85)	66.8 (54-85)	0.71*
CIMT (mm), median (min-max)	0.72 (0.47-0.87)	0.56(0.45-0.81)	0.0001**

BMI: body mass index; CIMT: carotid intima–media thickness.

*Pearson’s chi-squared test or Fisher’s exact test; **Mann–Whitney *U*-test.

According to the KDIGO classification, 58.7% (n = 37) of the patients were stage I AKI, 36.5% (n = 23) were stage II and 4.8% (n = 3) were stage III. Renal failure requiring dialysis developed in two of three patients with stage III AKI.

Pre-operative and early postoperative blood analysis and haematological parameters of the patients are summarised in Table 2. Pre-operative CRP (p = 0.0001), ESR (p = 0.0001), PLR (p = 0.0001) and NLR (p = 0.0001) were significantly different between the groups. Postoperative first-, third- and seventh-day CRP (p = 0.0001), postoperative first-, third- and seventh-day ESR (p = 0.0001), postoperative first-, third- and seventh-day PLR (p = 0.0001), and postoperative first-, third- and seventh-day NLR (p = 0.0001) were significantly different between the groups.

**Table 2:** Pre-operative and early postoperative blood results and haematological parameters of patients

*Haematological parameters*	*Group I AKI (n = 63) Median (min-max)*	*Group II non-AKI (n = 174) Median (min-max)*	*p-value*
Creatinine (mg/dl)			
Pre-operative	0.68 (0.42-1.42)	0.65 (0.37-1.41)	0.56*
Urea (mg/dl)			
Pre-operative	21.9 (12-39)	22.1 (14-39)	0.93*
eGFR (ml/min/1.73 m²)			
Pre-operative	99.0 (60-147)	98.8 (55-158)	0.71*
HbA16 (%)			
Pre-operative	5.8 (4.5-9.8)	5.7 (4.3-11.2)	0.18*
LDL-C(mg/dl)			
Pre-operative	118.6 (89-156)	119.0 (88-165)	0.59*
Haemoglobin (mg/dl)			
Pre-operative	13.3 (10.5-17.1)	13.7 (10.1-16.5)	0.36*
Postoperative day 1	8.8 (7.4-12.0)	8.9 (7.4-12.9)	0.68*
Haematocrit (%)			
Pre-operative	39.2 (30.5-48.9)	40.8 (30.6-48.9)	0.60*
Postoperative day 1	27.7 (24.3-35.8)	28.2 (23.8-38.4)	0.77*
Uric acid (mg/dl)			
Pre-operative	6.0 (2.3-9.7)	5.7 (2.2-9.1)	0.12*
Postoperative day 1	5.7 (4.5-9.8)	5.8 (4.3-10.8)	0.78*
Leukocyte count (X 103	cells/ul)		
Pre-operative	8.1 (5.0-10.9)	7.9 (4.1-10.9)	0.61*
Postoperative day 1	13.6 (5.8-22.4)	12.8 (6.1-30.4)	0.16*
Postoperative day 3	11.6 (5.1-26.4)	11.2 (4.6-25.7)	0.24*
Postoperative day 7	10.1 (5.2-24.6)	9.7 (4.3-25.1)	0.06*
CRP (mg/l)			
Pre-operative	1.79 (0.17-4.51)	0.51 (0.16-2.51)	0.0001 *
Postoperative day 1	32.5 (20.3-51.2)	27.4 (18.4-39.2)	0.0001 *
Postoperative day 3	45.7 (23.4-74.2)	28.1 (18.3-51.7)	0.0001 *
Postoperative day 7	19.4 (7.9-47.8)	9.5 (1.1-39.2)	0.0001 *
ESR (mm/h)			
Pre-operative	13.2 (2-19)	8.1 (2-19)	0.0001 *
Postoperative day 1	42.3 (13-61)	30.2 (10-74)	0.0001 *
Postoperative day 3	60.5 (32-88)	40.4 (23-81)	0.0001 *
Postoperative day 7	37.8 (18-51)	26.6 (16-50)	0.0001 *
PLR			
Pre-operative	152.7 (119.2-163.8)	135.2 (98.7-156.5)	0.0001 *
Postoperative day 1	158.7 (103.6-198.6)	128.8 (85.8-187.8)	0.0001 *
Postoperative day 3	152.8 (107.6-194.4)	124.4 (86.4-181.3)	0.0001*
Postoperative day 7	145.7 (95.4-185.8)	124.8 (68.7-196.9)	0.0001 *
NLR			
Pre-operative	5.2 (2.3-6.2)	3.5 (1.5-5.9)	0.0001 *
Postoperative day 1	6.6 (3.9-9.7)	5.1 (2.1-8.4)	0.0001 *
Postoperative day 3	6.1 (2.9-9.7)	4.9 (1.7-9.5)	0.0001 *
Postoperative day 7	5.2 (2.1-8.6)	3.8 (1.3-7.9)	0.0001 *

CRP: C-reactive protein; ESR: erythrocyte sedimentation rate; PLR: plateletto-

lymphocyte ratio; NLR: neutrophil-to-lymphocyte ratio; eGFR: estimated

glomerular filtration rate; HbA1c: glycosylated haemoglobin; LDL-C: low-density

lipoprotein cholesterol.

*Mann–Whitney U-test.

The intra-operative and postoperative data of the patients are shown in Table 3. Intubation time (p = 0.0001), stay in ICU (p = 0.0001) and length of hospital stay (p = 0.0001) were significantly different between the groups. Neurological event rate (transient ischaemic event, speech disorder, hemiplegia or hemiparesis) within the first 72 hours postoperatively was different between the groups [group 1; two (3.2%) patients, group 2; five (2.9%) patients; p = 0.60]. Likewise, in-hospital mortality following the first 72 postoperative hours occurred in one (1.6%) patient in group 1 and in two (1.1%) patients in group 2, which showed no statistical difference between the groups (p = 0.79).

**Table 3:** Intra-operative and postoperative data of the patients

	*Group I AKI (n = 63)*	*Group II non-AKI (n=174)*	
*Characteristics*	*Median (min-max)*	*Median (min-max)*	*p-value*
Aortic cross-clamping time (min)	54.5 (20-78)	56.1 (21-82)	0.81**
Cardiopulmonary bypass time (min)	85.5 (46-114)	86.5 (42-117)	0.68**
Number of distal anastomoses	3.22 (1-5)	3.30 (1-5)	0.64**
Amount of drainage (ml)	314 (200-1150)	317 (150-1200)	0.43*
Intubation time (h)	6.5 (4-12)	5.5 (3-11)	0.0001**
Stay in the ICU (h)	38.9 (18-96)	20.8 (17-87)	0.0001**
Total duration of hospital stay (days)	8.3 (5-16)	5.6 (5-11)	0.0001**
Use of inotropic support, n (%)	4 (6.3	16 (9.2%)	0.49*
Use of blood products, n (%)	29 (46.0)	74 (42.5)	0.63*

*Pearson’s chi-squared test or Fisher’s exact test; **Mann–Whitney U-test.

The results of univariate and multivariate regression analysis of patients who developed AKI in the early postoperative period are shown in Tables 4 and 5. In multivariate analysis, the variables that were found to be statistically significantly associated with postoperative AKI in univariate analysis were increased CIMT (p = 0.005), pre-operative CRP (p = 0.001), ESR (p = 0.005), NLR (p = 0.0001) and PLR (p = 0.0001); increased postoperative seventh-day CRP (p = 0.04), postoperative firstday PLR (p = 0.0001), postoperative seventh-day ESR (p= 0.02), and intubation time (p = 0.02). These were found to be independent predictors of early postoperative AKI. The result of the analyses showed not only that the regression model was significant [F (18, 218) = 57.09, p < 0.0001] but also that 81% (R2 adjusted = 0.81) of the variance of postoperative AKI, as the dependent variable, was expressed by the independent variables.

**Table 4:** Univariate and multivariate regression analysis of patient’s characteristics and pre-operative risk factors for postoperative AKI

		*Postoperative*		*AKI*	
*Variables*	*Unadjusted OR (95% CI)*	*p-value*	*R2*	*Adjusted OR (95% CI)*	*p-value*
Gender	1.36 (0.71-2.60)	0.36		-	
Age	1.03 (0.99-1.07)	0.11		-	-
Ejection fraction (%)	1.01 (0.97-1.04)	0.87		-	
Diabetes mellitus	0.90 (0.49-1.64)	0.73		-	
Hypertension	1.03 (0.56-1.89)	0.93		-	
Hyperlipidaemia	0.85 (0.47-1.52)	0.57		-	
Smoking	1.60 (0.89-2.88)	0.12		-	
BMI (kg/m²)	1.02 (0.94-1.10)	0.67		-	
CIMT (mm)	2.27 (1.82-2.71)	0.0001	0.30	4.04 (1.23-6.85)	0.005
Pre-operative creatinine (mg/dl)	1.39 (0.48-4.02)	0.54		-	
Pre-operative urea (mg/dl)	0.99 (0.93-1.06)	0.91		-	
Pre-operative uric acid (mg/dl)	1.18 (0.94-1.46)	0.15		-	
Pre-operative LDL-C (mg/dl)	0.99 (0.97-1.01)	0.54		-	
Pre-operative CRP (mg/l)	3.24 (2.79-3.70)	0.0001	0.46	6.90 (2.70-11.10)	0.001
Pre-operative PLR	2.00 (1.60-2.40)	0.0001	0.32	2.27 (1.14-3.40)	0.0001
Pre-operative NLR	2.66 (2.26-3.06)	0.0001	0.43	3.22 (1.46-4.98)	0.0001
Pre-operative haemoglobin (mg/dl)	0.92 (0.77-1.11)	0.39		-	
Pre-operative haematocrit (%)	0.97 (0.90-1.04)	0.41		-	
Pre-operative leucocyte (X 103 cells/ul)	0.99 (0.98-1.00)	0.44		-	
Pre-operative HbA (%)	1.17 (0.97-1.41)	0.11		-	
Pre-operative eGFR (ml/min/1.73 m²)	1.00 (0.98-1.02)	0.82		-	
Pre-operative ESR (mm/h)	1.31 (1.20-1.42)	0.0001	0.19	1.13 (0.87-1.45)	0.005

CRP: C-reactive protein; LDL-C: low-density lipoprotein cholesterol; ESR:

erythrocyte sedimentation rate; PLR: platelet-to-lymphocyte ratio; NLR: neutrophil-

to-lymphocyte ratio; BMI: body mass index; eGFR: estimated glomerular

filtration rate; HbA1c: glycosylated haemoglobin.

**Table 5:** Univariate and multivariate regression analysis of operative and early postoperative risk factors for postoperative AKI

		*Postoperative AKI*		
*Variables*	*Unadjusted OR (95% CI)*	*p-value R2*	*Adjusted OR (95% CI)*	*p-value*
Haemoglobin post- operative day 1	0.95 (0.74-1.23)	0.72	-	-
Haematocrit post- operative day 1	0.99 (0.90-1.09)	0.88	-	-
Uric acid postoperative day 1	0.97 (0.79-1.20)	0.79	-	-
CRP postoperative day 1	1.20 (1.13-1.27)	0.0001 0.17	0.89 (0.69-1.15)	0.47
CRP postoperative day 3	1.22 (1.16-1.28)	0.0001 0.46	1.18 (1.00-1.38)	0.11
CRP postoperative day 7	1.34 (1.24-1.45)	0.0001 0.38	1.11 (0.84-1.48)	0.04
PLR postoperative day 1	1.10 (0.90-1.30)	0.0001 0.34	2.03 (1.34-2.72)	0.0001
PLR postoperative day 3	1.21 (0.95-1.47)	0.0001 0.29	0.98 (0.94-1.02)	0.06
PLR postoperative day 7	0.70 (0.40-1.00)	0.0001 0.12	0.97 (0.93-1.01)	0.30
NLR postoperative day 1	1.52 (1.17-1.87)	0.0001 0.24	1.12 (0.84-1.40)	0.25
NLR postoperative day 3	1.06 (0.67-1.44)	0.0001 0.11	0.92 (0.86-0.98)	0.77
NLR postoperative day 7	1.35 (0.97-1.73)	0.0001 0.17	0.96 (0.92-1.00)	0.29
ESR postoperative day 1	1.08 (1.05-1.11)	0.0001 0.14	0.96 (0.83-1.11)	0.53
ESR postoperative day 3	1.16 (1.11-1.20	0.0001 0.39	1.16 (0.95-1.41)	0.17
ESR postoperative day 7	1.19 (1.13-1.25)	0.0001 0.25	1.21 (0.99-1.49)	0.02
Aortic cross-clamping time	1.00 (0.98-1.02)	0.90	-	-
Number of distal anastomoses	0.96 (0.72-1.27)	0.77	-	-
CPB time	1.00 (0.98-1.02)	0.87	-	-
Intubation time	1.53 (1.25-1.86)	0.0001 0.09	3.63 (1.22-10.82)	0.02
Use of blood products	1.15 (0.65-2.06)	0.63	-	-
Use of inotropic support	0.72 (0.23-2.25)	0.57	-	-
Amount of drainage	1.00 (0.99-1.01)	0.51	-	-

CRP: C-reactive protein; CPB: cardiopulmonary bypass; ESR: erythrocyte sedimentation

rate; NLR: neutrophil-to-lymphocyte ratio; PLR: platelet-to-lymphocyte ratio.

**Fig. 1:**
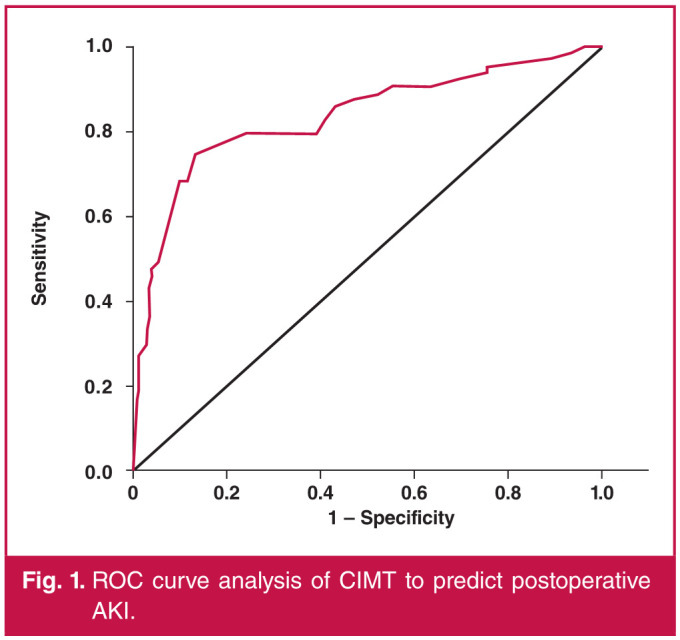
ROC curve analysis of CIMT to predict postoperative AKI.

The ROC curves for CIMT were in connection with postoperative AKI following isolated CABG (Fig. 1). The area under the curve (AUC) for CIMT was 0.834 (95% CI: 0.768– 0.900; p = 0.0001). Using a cut-off value of 0.66 mm, CIMT predicted postoperative AKI with a sensitivity of 79.4% and specificity of 75.9%.

## Discussion

In this study, we aimed to determine the association between pre-operative CIMT of the patients operated on for isolated CABG and early postoperative AKI. To our knowledge, this study is one of the few on this subject.

The optimal cut-off value of 0.66 mm for the CIMT was found to be a strong and independent predictor of AKI. Moreover, elevated CRP level and ESR, PLR and NLR in the pre- and early postoperative periods were found to be associated with AKI. Therefore, pre-operative CIMT may be a useful, inexpensive and novel marker to predict AKI in the early postoperative period of isolated CABG. Pre- and early postoperative CRP, ESR, PLR and NLR may also be seen as early markers.

Major adverse renal and cardiac events still exist as potential threats following cardiac surgery, despite the advances in surgical technique, anaesthetic management and supportive medical treatment.^21^ AKI is a common postoperative complication of cardiac surgery, associated with prolonged hospital stay and increased early morbidity and mortality rates, even for patients who do not progress to renal failure.^22^ The pathophysiology of AKI following cardiac surgery is complex and multifactorial, including renal ischaemia–reperfusion injury, exogenous and endogenous toxins, use of radiocontrast media, neurohormonal activation, metabolic factors, hypoproteinaemia, inflammation and oxidative stress.^23^

After cardiac surgery, AKI is seen at a rate of 5–48% and is associated with a 50% increase in early postoperative mortality.^24^ Hobson et al. reported AKI in 43% of cardiac surgery patients during in-hospital follow up.^25^ For standardisation, patients operated on for only isolated CABG using standard CPB were enrolled in our study, therefore postoperative AKI and acute renal failure occurred in 26.6 and 4.8% of the patients, respectively, which is in accordance with the literature.

AKI incidence following cardiac surgery depends on its definition. The Risk, Injury, Failure, Loss of kidney function, and End-stage kidney disease (RIFLE) classification, Acute Kidney Injury Network (AKIN) criteria and KDIGO stages, are all practical predictors of AKI after CABG and/or heart valve operations.^19^,^26^ In our study, AKI was defined according to the KDIGO criteria.

Since the role of inflammation on AKI has been shown in numerous studies, biomarkers of cellular inflammation, such as NLR and PLR, which are inexpensive and routinely tested, have been used frequently.^15^ Parlar et al. showed that elevated pre- and postoperative NLR and PLR levels were independently related to early postoperative AKI.^16^ In our study, we noted that pre- and postoperative NLR and PLR values were significantly higher in patients with AKI.

Atherosclerosis is a chronic, multifactorial disease, generally affecting the entire arterial system.^2^ Since inflammation acts as a common base for physiological and pathological changes throughout the initiation and evolution of atherosclerosis, there has been vast experimental and clinical evidence regarding atherosclerosis as a chronic inflammatory disease.^27^ Leucocytes, mediators of host defence and inflammation, have been demonstrated in the earliest lesions of atherosclerosis, in both animal experiments and humans.^28^

CIMT, which shows both endothelial dysfunction and diffuse atherosclerosis, is one of the methods to determine the atherosclerotic process during the asymptomatic period.^2^ CIMT determination is a non-invasive, low-cost, easy and repeatable procedure to evaluate atherosclerosis.^3^ CIMT is not only a predictor of atherosclerosis, it also predicts myocardial infarction, stroke and future cardiovascular events.^4^ SahBandar et al. reported a strong correlation between inflammatory monocyte counts and CIMT.^29^ Significant correlations between IL-6 and CIMT in patients with the metabolic syndrome were also reported.^30^

Despite many studies, data regarding the relationship between CIMT and other indices of vascular injury are limited and controversial.^3^ Besir et al. emphasised that CIMT, which is correlated with major risks for atherosclerosis, such as age, visceral fatty tissue mass, fasting glucose level, and total and low-density lipoprotein cholesterol (LDL-C) levels in healthy adults, is a valuable test for the measurement of subclinical atherosclerosis and cardiovascular risk potential.^31^ A diffuse increase in CIMT is a predictive factor for arterial plaque formation. Genetic and acquired factors other than age are also considered to direct this progression; therefore, this reflects the relationship between diffuse intimal thickening and atheromatous lesions.^20^ In our study, the difference in CIMT in the AKI group was statistically significant, unlike the age difference.

Recently, several prospective clinical studies have shown that even modest elevations in baseline CRP levels could predict cardiovascular events.^32^ CRP is the fastest elevated acute-phase reactant following inflammation or injury and is the fastest depleted during healing.^33^ CRP is considered an independent predictor of intima–media thickness.^32^ Shacham et al. reported on patients with ST-segment elevation myocardial infarction treated with primary percutaneous coronary intervention (PCI). The presence of a high-sensitivity CRP (hs-CRP) level > 9 mg/l prior to PCI was an independent risk factor for AKI.^34^

Zoccali et al. reported on patients with initially normal CIMT levels that elevated CRP was related to changes in the CIMT. The interaction between these two resulted in a single independent predictor of intimal lesion progression.^33^ Likewise, in our study, pre- and postoperative CRP elevation was a direct indicator of systemic inflammation in patients with postoperative AKI. The relationship between elevated CIMT and postoperative AKI suggested that inflammatory processes were operative on AKI. Since atherosclerosis is a chronic inflammatory disease, the lack of studies in the literature regarding the relationship between CIMT and postoperative AKI was the main reason to perform this study.

ESR is a simple and inexpensive laboratory test that measures aggregation tendency of red blood cells that were shown to be elevated in tissue injury and necrosis, and a risk factor for atherothrombotic cardiovascular disease.^35^ Singh et al. reported that ESR was correllated with atherosclerosis markers and might carry important prognostic information about later catastrophic events, such as stroke, myocardial infarction and AKI, which verified the strength of the inflammatory response related to carotid artery atherosclerosis.^36^ Early diagnosis of AKI, which means awareness of a surreptitious onset of postoperative acute renal failure, may be facilitated by determining ESR and CRP levels.^22^ Likewise, high pre-operative and early postoperative levels of ESR were related to AKI in our study. Also, the CIMT of patients with a high ESR was found to be significantly increased.

The unpredictability of AKI and progression to chronic kidney disease due to ineffective treatment make AKI a global problem to be solved.^27^ Determining renal function disorder in the postoperative period may enable the detection of patients with increased long-term risk in order to take preventative measures during follow up.^9^ Very little evidence has been obtained from randomised studies regarding protection or supporting specific interventions to prevent AKI.^25^ Novel biomarkers of kidney injury may identify subclinical diagnosis, severity and prognosis of AKI following cardiac surgery and enable interventions for the reduction of incidence of and protection from AKI.^11^ The primary aim of our study was to determine the predictive value of pre-operative CIMT as a marker of possible AKI in the early postoperative period. An independent correlation was found.

Experimental evidence shows that initiation and continuation of AKI arise from inflammation-mediated injury.^15^ AKI diagnosis is delayed until at least the extension phase, when alterations in endothelial leukocyte interactions may be a prominent activity.^37^ Recent studies have shown the relationship between estimated glomerular filtration rate (eGFR) and subclinical atherosclerosis.^4^ Ito et al. reported that not the level of diabetic nephropathy graded by urinary albumin discharge, but eGFR was correlated with CIMT in type 2 diabetes.^38^ These alterations may relate to peritubular capillary loss in the long term, which can be preserved by experimental treatment during the early phases of injury and repair.^10^

CPB induces systemic inflammatory response syndrome, negatively affecting renal perfusion. In a study where CABG with CPB was compared with CABG on a beating heart with CPB support, no difference was found in terms of postoperative AKI.^39^ Our study population included only patients operated on for isolated CABG on standard CPB. Although there were no statistically significant differences between the AKI and non-AKI groups regarding the duration of CPB, the relationship found between AKI and CIMT, PLR, NLR, ESR and CRP, regardless of other risk factors, suggested that systemic inflammation may be operative both on CIMT increase and the occurrence of AKI. Therefore, if AKI occurred in patients, it might be foreseen that they were exposed to systemic inflammatory processes. CIMT levels found in this study might be a marker for subclinical systemic inflammation already in the pre-operative period, as well as for postoperative AKI.

Inflammation playing a role in the aetiopathogenesis of AKI revives the use of CIMT as a predictor to prevent this injury.^27^ Lorenz et al. showed in their study that elevated CIMT was an independent marker of vascular events, and this relationship was stronger, particularly among younger individuals. This study was well standardised in terms of risk stratification, ultrasonographic examination and CIMT measurements.^5^ In another study in a Chinese population, systemic arterial changes were shown to begin in the early phase of chronic kidney disease.

It was reported that traditional factors might be operative on elevation of CIMT, however, the relationship between elevated CIMT and patient prognosis should be examined in depth.^17^ A correlation between LDL-C and CIMT was also mentioned to exist when adjusted to traditional risk factors such as advanced age, male gender, smoking and family history of cardiovascular disease.^6^ Likewise, among the population operated on for isolated CABG and standardised with strict exclusion criteria in our study, statistically significantly higher pre-operative CIMT in the AKI group suggested a common cause for both AKI and CIMT, such as systemic inflammation. Our study groups were statistically similar in terms of age, gender, smoking and pre-operative LDL-C levels.

One of the few studies on a relationship between CIMT and postoperative AKI in patients operated on for CABG was from Onk et al., which was different from our study in terms of exclusion of patients who died and selection method of the groups.^18^ In our study, the real-life data of a stable, homogeneous and well-selected group was sought completely in terms of inflammatory response over a six-year period. CIMT was found as an independent predictor of postoperative AKI, together with pre-operative CRP, ESR, PLR and NLR. Additionally, a greater increase in the markers of postoperative inflammation in the AKI group supports the relationship between AKI and inflammation. With these results, risk for AKI may be defined by gaining information about the pre-operative inflammatory status of the patient, using CIMT.

Unlike other suggested markers, the potential advantage of CIMT is that it is radiologically measured and is not a blood test; therefore, it will remain unaffected by temporary disturbances of blood analysis and inflammatory markers due to pre-operative acute events. From the relationship found between pre-operative CIMT and postoperative AKI, sufficient time may be obtained for both surgical and pharmacological planning and modification in the pre-operative period to prevent postoperative AKI.

## Limitations of the study

First, this was a retrospective, observational study so causal inference cannot be assumed, and it was subject to bias from several factors. Although we attempted to control selection bias with multivariate statistical methods and adjusted for risk factors that affect postoperative AKI, we could not eliminate the potential for residual confounding. In the absence of a comparable prospective database of AKI after cardiac surgery, a retrospective study with compensatory statistical methods is a reasonable approach.

Second, this was a single-centre study with a relatively small sample size. Third, we did not have data about the severity of the disease at the time of admission to ICU. Even though there may have been inaccuracy and differences in data, we assumed that these were distributed randomly and would not result in an important bias in our results.

Fourth, we did not have access to information regarding the subsequent medical care of these patients. Fifth, CIMT was measured at a single time point. In studies where progression of CIMT was taken into account, which is suggested to vary in different segments of the carotid arteries, finding similar results becomes unclear if changes in internal carotid artery intima– media thickness (I-IMT) compared to CCA-IMT in time were considered.

Sixth, when CCA-IMT and I-IMT are compared to assess prediction of AKI risk following cardiac surgery, use of a more modern ultrasound technology with smaller variability and enhanced resolution may result in different values. Seventh, non-traditional risk factors for increase in CIMT were not assessed in this study. Finally, although we could not establish a causal relationship in this study, the results may have some clinical implications if confirmed by large-scale prospective studies. Therefore, CIMT may not lose its predictive power in terms of AKI prediction in larger patient groups and patients with acute pre-operative events.

## Conclusion

We observed that elevated CRP, ESR, PLR, NLR and CIMT were independent predictors of early postoperative AKI following isolated CABG. These findings suggest that the factors proven to be related to inflammation may predict adverse events following CABG. As a measurable morphological change that is related to both AKI and the inflammatory burden, CIMT may be considered a predictor that can be used more widely than pre-operative blood tests. We hope that our study may be useful in providing more evidence on the effectiveness and applicability of this marker.
